# Remote-Controlled Gene Delivery in Coaxial 3D-Bioprinted Constructs using Ultrasound-Responsive Bioinks

**DOI:** 10.1007/s12195-024-00818-x

**Published:** 2024-10-27

**Authors:** Mary K. Lowrey, Holly Day, Kevin J. Schilling, Katherine T. Huynh, Cristiane M. Franca, Carolyn E. Schutt

**Affiliations:** 1 Biomedical Engineering Department, Oregon Health and Science University, Portland, OR 97201 USA; 2 Cancer Early Detection Advanced Research Center, Knight Cancer Institute, Oregon Health and Science University, Portland, OR 97201 USA; 3 Knight Cancer Precision Biofabrication Hub, Knight Cancer Institute, Oregon Health and Science University, Portland, OR 97201 USA; 4 Department of Oral Rehabilitation and Biosciences, School of Dentistry, Oregon Health and Science University, Portland, OR 97201 USA

**Keywords:** Microbubbles, Sonoporation, Biomaterials, Coaxial 3D bioprinting, Gene delivery, Focused ultrasound, Controlled delivery, Bioink, Ultrasound

## Abstract

**Introduction:**

Coaxial 3D bioprinting has advanced the formation of tissue constructs that recapitulate key architectures and biophysical parameters for in-vitro disease modeling and tissue-engineered therapies. Controlling gene expression within these structures is critical for modulating cell signaling and probing cell behavior. However, current transfection strategies are limited in spatiotemporal control because dense 3D scaffolds hinder diffusion of traditional vectors. To address this, we developed a coaxial extrusion 3D bioprinting technique using ultrasound-responsive gene delivery bioinks. These bioink materials incorporate echogenic microbubble gene delivery particles that upon ultrasound exposure can sonoporate cells within the construct, facilitating controllable transfection.

**Methods:**

Phospholipid-coated gas-core microbubbles were electrostatically coupled to reporter transgene plasmid payloads and incorporated into cell-laden alginate bioinks at varying particle concentrations. These bioinks were loaded into the coaxial nozzle core for extrusion bioprinting with CaCl_2_ crosslinker in the outer sheath. Resulting bioprints were exposed to 2.25 MHz focused ultrasound and evaluated for microbubble activation and subsequent DNA delivery and transgene expression.

**Results:**

Coaxial printing parameters were established that preserved the stability of ultrasound-responsive gene delivery particles for at least 48 h in bioprinted alginate filaments while maintaining high cell viability. Successful sonoporation of embedded cells resulted in DNA delivery and robust ultrasound-controlled transgene expression. The number of transfected cells was modulated by varying the number of focused ultrasound pulses applied. The size region over which DNA was delivered was modulated by varying the concentration of microbubbles in the printed filaments.

**Conclusions:**

Our results present a successful coaxial 3D bioprinting technique designed to facilitate ultrasound-controlled gene delivery. This platform enables remote, spatiotemporally-defined genetic manipulation in coaxially bioprinted tissue constructs with important applications for disease modeling and regenerative medicine.

**Supplementary Information:**

The online version contains supplementary material available at 10.1007/s12195-024-00818-x.

## Introduction

Coaxial 3D bioprinting has been established as a versatile fabrication method for tissue constructs due to its ability to produce complex tissue-like architectures containing multiple cell types and to mimic biophysical parameters of the matrix microenvironment. Compared to 2D culture, 3D culture better recapitulates in-vivo properties such as cell–cell and cell-matrix interactions, protein expression, and enzyme activity [1]. 3D model systems are also more amenable to cellular imaging than in-vivo models [2, 3]. 3D bioprinting provides advantages for creating cell-laden architectures, such as spatial control over matrix properties [4], biochemical factors [5], and cell type [6].

Coaxial bioprinting, which utilizes a nozzle with multiple concentric orifices, has garnered significant interest as an extrusion-based bioprinting method, as it allows for precisely controlled simultaneous bioprinting of multiple bioinks to form complex constructs [7]. By having a core material and outer sheath material that can each be printed with different parameters, coaxial bioprinting enables the co-printing of hydrogel bioinks with different properties, such as those with complementary mechanical or biochemical features. The concentric nozzle design also allows for single-step crosslinking by extrusion of the bioink from the core and the crosslinking agent from the outer sheath. Conversely, the crosslinker can be extruded from the core with bioink in the sheath to form hollow fibers [8]. Coaxial bioprinting can also incorporate sacrificial materials in a single printing step, such as a sacrificial core to form a hollow tubular structure [7–9]. These features make coaxial bioprinting an important method for creating 3D tissue constructs, as it enables the production of more physiologically relevant tissue architectures, such as vasculature [10–13] as well as zonal cartilage organization and intestinal villus structures [7, 14, 15].

The ability to genetically manipulate cells within coaxially bioprinted tissue constructs is an essential tool for modeling dynamic changes in cell signaling and enables the perturbation of cell behaviors. This has utility for stimulating intercellular communication, growth factor release, and stem cell differentiation in tissue constructs [16] to direct cell function in the 3D microenvironment, and can also be used to model disease states. Spatiotemporal control over gene delivery is critical to allow cell structures within constructs to self-assemble and mature, forming cell-cell connections and signaling interactions before initiating genetic changes in selected cells or tissue regions. This is an important order of events to model genetic changes within fully formed tissue structures. Remote genetic manipulation also enables the ability to transform a small subset of user-defined cells within the established tissue construct which is different from transforming all the cells all at once. This allows researchers to study how these transformed cells interact with the normal cells around them, in a biomimetic tissue environment. An important application for controlled genetic manipulation in 3D constructs includes disease modeling including cancer development, which involves genetic and epigenetic changes [17] in a subset of cells within a mature tissue that has established connections and intercellular signaling.

However, achieving spatial and temporal control over genetic manipulation of cells within coaxially-bioprinted constructs is a challenge because scaffold matrices hinder the diffusion of traditional transfection vectors, making it difficult to control their localization. Current methods for gene delivery used in 2D culture, such as commonly used viral transduction, lack the ability to both spatially and temporally control the genetic manipulation of cells within 3D constructs [18]. Additionally, biomaterial scaffolds present challenges for viral transduction, as thick constructs and crosslinked filaments can hinder virus diffusion, which can result in uneven gene delivery [19]. Chemical transfection methods allow for cross-membrane DNA delivery, but lack spatiotemporal control of genetic manipulation within the 3D tissue construct [20]. Gene activated matrices (GAM) are a platform for localized and sustained gene delivery in biofabricated 3D constructs, and have shown potential for directing gene expression in 3D scaffolds [21, 22]. In these systems, nucleic acids are incorporated into the construct matrices and the GAM platform facilitates the sustained release of nucleic acids to embedded cells. GAM-based DNA delivery has also been shown to be effective at delivering DNA to cells in-vivo after implantation into tissue [23, 24]. These GAM-based strategies rely on passive diffusion or cellular motility for the incorporated DNA to interact with cells which does not allow for precise control over when and where the DNA is delivered. To address the challenge of achieving spatiotemporal control over gene delivery, we have designed an ultrasound-responsive platform that enables user-defined control over DNA delivery in combination with bioprinted architectures. This allows for an established print to be genetically manipulated remotely and on-demand. Genetic manipulation in tissue constructs has been used to elucidate the function and impact of specific genes. It has been shown that gene functionality and cellular gene expression depends on both the spatiotemporal and architectural context of the matrix [25–29]. As such, the ability to remotely control genetic manipulation for precise spatial and temporal control within tissue constructs is critical for functional characterization of genetic impact on cellular processes and phenotypes. However, current strategies are limited in their ability to create a relevant 3D architecture containing multiple cell types and lack the function of spatial and temporal gene delivery.

Focused ultrasound has several desirable properties as an activating stimulus for triggering gene delivery in tissue including its multi-centimeter tissue penetration depth, biocompatibility, and ease of remote application [16, 30, 31]. A low-intensity focused ultrasound beam can be used in combination with ultrasound-responsive gene delivery particles, such as gas-core microbubbles [31, 32], which are frequently injected intravenously to facilitate gene delivery within a localized tissue region of interest. [33–37] These microbubbles contain a gas core, typically a mixture of air and low solubility perfluorocarbon gas, stabilized by a lipid monolayer [38–41]. Plasmid DNA can be electrostatically coupled to the surface of microbubbles that contain cationic lipids in the lipid monolayer coating [32, 33, 35, 36]. When focused ultrasound is applied, the microbubbles rapidly oscillate, cavitate, and release their DNA payload [42–44]. The microbubble and ultrasound interaction leads to an effect known as sonoporation [42, 45–47], where nearby cell membranes are transiently disrupted to allow nucleic acid payload to enter the cell, leading to genetic manipulation of the cells in the focal zone. The ultrasound can be spatially focused to a region on the order of a cubic millimeter achieving spatiotemporal control over the ultrasound trigger and minimizing off-target DNA delivery [42, 48]

Focused ultrasound has several advantageous characteristics compared to other external stimuli, such as light. Ultrasound has a multi-centimeter penetration depth through cell-dense tissue constructs [30] in contrast to light which is in the range of multi-millimeter scale penetration [49, 50]. Near infrared (NIR) light, while capable of deeper penetration, has difficulty being focused through a highly scattering medium like a dense tissue construct, while ultrasound has much less scattering and can achieve tight focal zones.

**Fig. 1:**
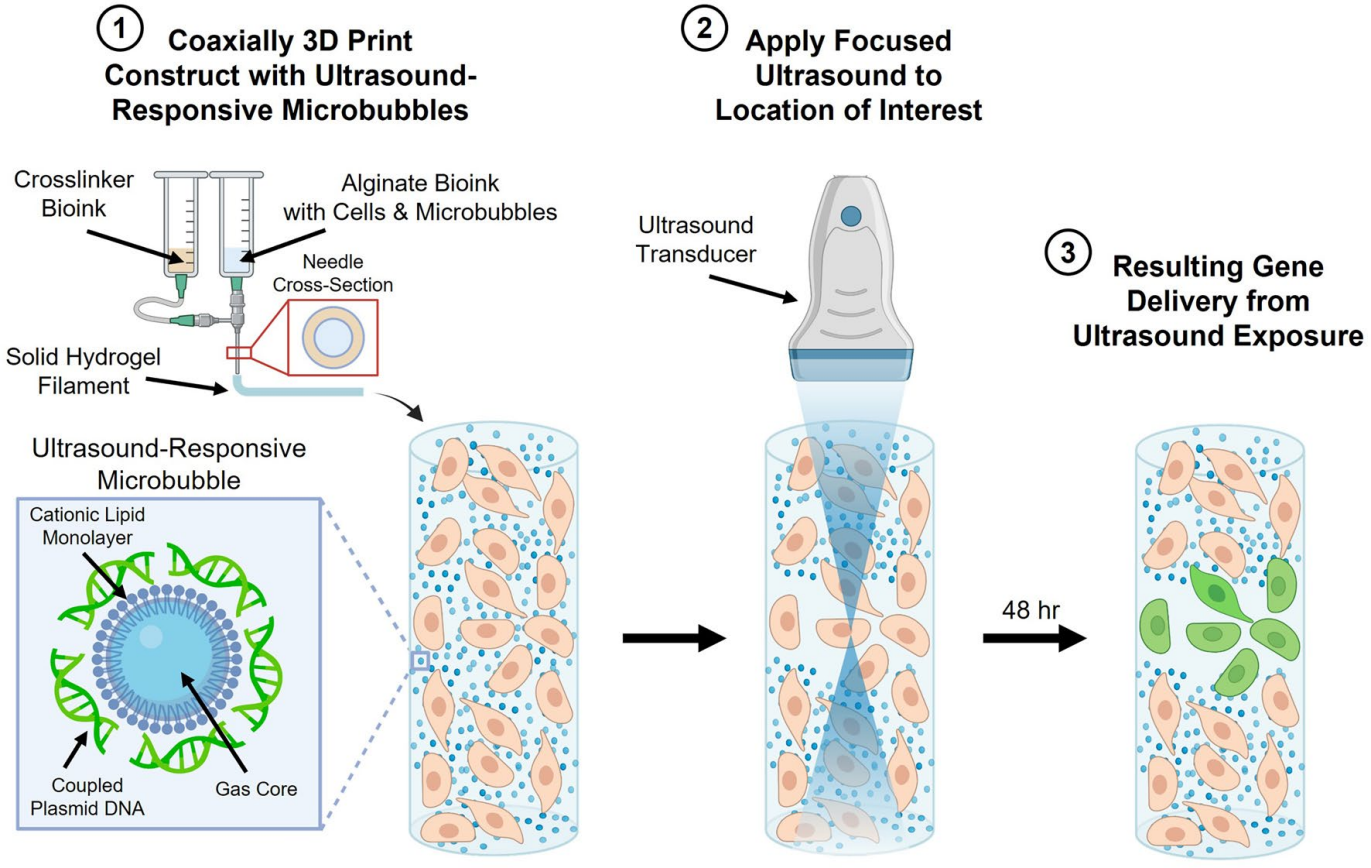
Coaxial 3D bioprinting of microbubble gene delivery vehicles. The schematic depicts coaxial bioprinting of a 3D construct containing cells and DNA-coupled ultrasound-responsive microbubbles. The construct is printed with a coaxial needle where the needle core contains sodium alginate bioink precursor solution, microbubble gene delivery vehicles, and cells. The sheath compartment contains calcium crosslinking agent thereby enabling printing of microbubble-containing crosslinked hydrogel filaments. Post-printing, focused ultrasound is applied to user-defined areas of the construct resulting in spatiotemporally controlled regions of gene delivery

Here we present a new coaxial bioprinting platform technology that leverages these ultrasound/microbubble interactions to address the current limitations of spatiotemporally controlling genetic manipulation in cell-seeded coaxial bioprinted constructs. We developed a coaxial bioprinting technique, using bioinks containing ultrasound-responsive gene delivery particles, that addresses the unique challenges of multiple fluids being extruded and mixing at the end of the nozzle to achieve microbubble distributions and stability that allows for spatial and temporal control of genetic manipulation (Fig. 1). We incorporate echogenic microbubble gene delivery particles and cells into a sodium alginate bioink precursor solution in the core of the coaxial needle, with calcium ion crosslinker in the sheath compartment. These solutions are simultaneously printed using the coaxial nozzle so that the alginate bioink is crosslinked upon extrusion to form alginate filaments containing cells and microbubbles. These filaments can be deposited with spatial control to form larger structures. Focused ultrasound can then be applied to a user-defined region of the coaxially bioprinted construct where the echogenic microbubbles oscillate and rupture inducing sonoporation and localized DNA delivery. This facilitates spatiotemporally controllable genetic transfection of cells only in the targeted region.

This paper demonstrates our newly developed method of coaxially bioprinting ultrasound-responsive microbubbles within cell-laden bioinks to enable spatiotemporal-controlled genetic manipulation in the constructs. We showcase our ability to bioprint high-viability cell-laden scaffolds and use focused ultrasound to modulate the size of the DNA delivery zone and number of genetically manipulated cells in a 3D construct. This new coaxial bioprinting platform enables remote-controlled genetic manipulation in printed tissue constructs and can be leveraged for future studies of genetic drivers of disease as well as to remotely guide tissue healing and regeneration through controlled gene expression.

## Materials and Methods

### Fabricating Ultrasound-Responsive Microbubbles

Cationic ultrasound-responsive microbubbles (µB) were utilized to facilitate ultrasound-responsive gene delivery within our bioprinted structures. These microbubble particles contain a gas core of a mixture of air and low-solubility perfluorocarbon gas, stabilized by a lipid monolayer. The cationic lipid surface of the microbubble allows for electrostatic coupling of plasmid DNA [32, 33, 35, 36, 42]. The microbubbles were fabricated prior to incorporation within bioinks.

Briefly, the lipids 18:0 PC (1,2-distearoyl-sn-glycero-3-phosphocholine, 27.2 g, Avanti Polar Lipids), 18:0 TAP (1,2-stearoyl-3-trimethylammonium-propane (chloride salt), 6.4 g, Avanti Polar Lipids), and 18:0 PEG2000 PE (1,2-distearoyl-sn-glycero-3-phosphoethanolamine-N-[methoxy(polyethylene glycol)-2000] (ammonium salt), 6.4 g, Avanti Polar Lipids) in powder form were combined in a glass scintillation vial and suspended in 1X DPBS without calcium or magnesium (–Ca/–Mg, 6 mL, Gibco). The solution was sonicated using a 3.4 mm sonication probe for 3 minutes at 90% intensity to thoroughly mix the lipids. Upon mixing, hydrophobic perfluorocarbon (PFC) gas (octafluoropropane, APF-N40HP (99.8%), FluoroMed) was sparged into the scintillation vial and sonicated at 90% intensity for 10 seconds, resulting in the formation of microbubbles with a PFC and air gas interior and lipid monolayer exterior including the cationic 18:0 TAP lipid. The microbubbles were rested at room temperature for 15 minutes and then added to a capped glass vial and centrifuged at 1500 RPM for 1 minute. This step caused the microbubbles to float and separate from the subnatant of DPBS buffer and excess lipid. This subnatant was then removed using a syringe with a 22G needle, and 2 mL of fresh 1X DPBS was added to the vial before tapping to mix and centrifuging again. This process was repeated for a total of five washes to remove excess lipids. The microbubbles were then resuspended in 300 µL of 1X DPBS, tapped to mix well, and counted to obtain a concentration of µB/mL using a hemocytometer and brightfield microscope.

### Coupling pCAG-GFP DNA Plasmid to Ultrasound-Responsive Microbubbles

pCAG-GFP plasmid was added at a ratio of 0.1 pg of pCAG-GFP plasmid DNA per microbubble. pCAG-GFP was a gift from Connie Cepko (Addgene plasmid # 11150; http://n2t.net/addgene:11150; RRID:Addgene_11150) [51]. After adding the required solution of plasmid DNA, the vial containing the microbubbles was shaken to thoroughly mix the plasmid and microbubble solution, and was mixed every 15 minutes for two hours. After the microbubbles were coupled with DNA, they were counted again via hemocytometer and brightfield microscope to determine a final concentration of the stock microbubble solution prior to incorporation into the bioinks. For visualization of coupled DNA, plasmid-coupled microbubbles were stained with YOYO-1 Iodide nucleic acid stain (Thermo Fisher Scientific) and washed with 1X DPBS prior to imaging. Overlaying the brightfield and fluorescence images of the microbubbles allows visualization of the DNA coupling, as fluorescent signal of the YOYO-1-stained DNA localizes to the surface of the microbubbles.

### Size Distribution of Ultrasound-Responsive Microbubbles

The ultrasound-responsive microbubbles were diluted at a factor of 1:200 and placed between two 1.5 glass coverslips before imaging on the Leica Thunder 3D cell culture widefield microscope imaging system. Microbubble imaging was performed using a 63X oil immersion lens. Microbubble size analysis was obtained by automated particle analysis performed in ImageJ. Briefly, image thresholding was applied to segment the microbubble particles and the automated fill holes and watershed functions were applied to the thresholded microbubble image. Automated particle sizing was performed on the segmented microbubbles by specifying a minimum pixel size and selecting a 0.8–1.0 circularity interval.

### Fabricating Bioinks for Coaxially Bioprinting Constructs

Sodium alginate is a naturally occurring polymer that is non-toxic and biodegradable, with a tunable range of mechanical properties [52–54]. Alginate is frequently utilized as a bioink [55] and concentrations of 2-6% w/v have been previously employed to simulate tissue environments [53, 56–58]. Alginate bioinks were prepared at 2% w/v, 4% w/v, and 6% w/v by adding powdered sodium alginate (3D Systems) to DI water (UltraPure™ DNase/RNase-Free Distilled Water, Invitrogen). These inks were vortexed to mix and then added to a bead bath at 60 °C for 15 minutes. The inks were vortexed again and stirred using a metal stirrer to break up large powder fragments before being placed in the bead bath for another 15 minutes. This process was repeated a minimum of three times, or until the alginate powder and water completely incorporated together. The alginate bioinks were then centrifuged at 1000 RPM for 5 min to remove any air bubbles that were incorporated during the mixing process. Alginate functionalized with RGD peptide (arginylglycylaspartic acid), which promotes cellular adhesion within the bioprint, [59], was prepared for printing the osteoblasts by diluting 10% w/v A-RGD (Cellink) with cell media for a final concentration of 4% w/v alginate.

Gelatin bioink with calcium chloride crosslinking agent was prepared by adding gelatin (gelatin from bovine skin Type B, Sigma Aldrich) to a solution of calcium chloride (CaCl_2_, 3D Systems) in DI water for a final concentration of 2% w/v gelatin, 2% w/v CaCl_2_. Gelatin is used to increase the viscosity of the crosslinker bioink, as it is challenging to control the flow of less viscous liquids when extrusion bioprinting. After crosslinking the alginate, the gelatin was dissolved by placing the print at 37 °C and removed by rinsing with fresh media. To prepare the gelatin ink, the ink was vortexed to mix and added to a bead bath at 85 °C for 5 min. The solution was vortexed again and stirred using a metal stirrer to break up large powder fragments before being placed back into the 85 °C bead bath for an additional 25 min. The ink was placed at 4 °C for 15 min, then placed in the bead bath at 85 °C for 30 min. This process was repeated for three total rounds of heating and cooling. After the final cycle, 2 mL of the gelatin bioink was pipetted into a 3 mL bioprinting syringe (Cellink). The solution was allowed to cool at room temperature overnight to partially solidify the gelatin for bioprinting ease.

### Incorporating Plasmid-Coupled Ultrasound-Responsive Microbubbles into Alginate Bioinks

Alginate bioinks were prepared as previously stated, and 400 µL of the respective bioink was added to a 3 mL bioprinting syringe (Cellink). Plasmid-coupled microbubbles were prepared as previously stated, and added to the 2%, 4%, and 6% w/v alginate bioinks at a final concentration of 2.34 × 10^9^ µB/mL, and stirred gently to avoid the incorporation of air bubbles using a 22G blunt-tip needle attached to a syringe.

### Incorporating Cells into Microbubble-Laden Alginate Bioinks

Human embryonic kidney (HEK293T, ATCC CRL-3216) cells were obtained at 80% confluency between P6-P16 from a T75 flask using TrypLE (TrypLE™ Express, Gibco) for lifting. They were then suspended in complete media (DMEM, high glucose, Gibco), 10% FBS (HyClone Characterized Fetal Bovine Serum CA Origin, Cytiva), and 1% pen-strep (penicillin-streptomycin, 10,000 µg/mL, Gibco) and concentrated to 3.85 × 10^7^ cell/mL in complete media.

Human osteoblast (hFOB 1.19, ATCC CRL-3602) cells were obtained at 80% confluency at P12 from a T75 flask using TrypLE (TrypLE™ Express, Gibco) to lift them. They were then suspended in complete media containing Human Osteoblast Media (Cell Applications, Inc.), 10% FBS (HyClone Characterized Fetal Bovine Serum CA Origin, Cytiva), and 1% pen-strep (penicillin-streptomycin, 10,000 µg/mL, Gibco) then concentrated to 3.85 × 10^7^ cell/mL in complete media.

The Cellink Bio X6 bioprinter was set to 37 °C to maintain viability of the cells. Upon preparation for bioprinting, 20 µL of the appropriate cells at 3.85 × 10^7^ cell/mL were added to the syringe containing the previously prepared solution of alginate bioink and ultrasound-responsive microbubbles. The cells were mixed in by stirring gently to avoid the incorporation of air bubbles using a 22G blunt-tip needle attached to a syringe. The loaded bioprinting syringe was then immediately loaded into the bioprinter at 37 °C.

### Bioprinting Cell-Laden Ultrasound-Responsive Alginate Bioinks

The Cellink Bio X6 bioprinter with DNA Studio 4.0 software was used throughout all experiments. The previously prepared syringe containing the cell-laden ultrasound-responsive alginate bioink was attached to the core inlet of a coaxial nozzle at 37 °C, and the previously prepared syringe containing the 2% gelatin in 2% CaCl_2_ was attached to the sheath inlet of the coaxial nozzle [20G inner diameter core, 16G outer diameter sheath (3D Systems)] at room temperature. The bioprinter was calibrated to touch the top of a glass slide and then moved upward 0.2 mm to give clearance space for the print to extrude. An 8-well dish (LabTek 8-well 1.0 cover glass bottom, LabTek) was needed for ultrasound manipulation of the bioprinted construct but caused a lack of clearance with the printing needle. This meant the construct was printed directly onto a slide and transferred to the 8-well dish after printing.

The bioinks were printed at 4 mm/s, with the alginate printing at 37 °C (for cell viability) in the range of 10–15 kPa, and the gelatin printing at room temperature in the range of 40–60 kPa. A pre-flow delay of − 400 ms and a post-flow delay of − 400 ms was used for all inks to aid print fidelity. The pre-flow delay allowed for the bioink to begin extruding briefly before the printer nozzle began moving, which aided the ability of the ink to be printed onto the slide without dragging the filament around. The post-flow delay ensured that the printing pressure was removed before completing the print to prevent smearing and account for nozzle leakage. Both of the flow delays were negative values, meaning that the pre-flow delay caused the ink to extrude before the nozzle began moving, and the post-flow delay caused the ink to stop extruding before the nozzle stopped. The alginate core containing the microbubbles and cells was surrounded by the 2% gelatin 2% CaCl_2_ sheath enabling the alginate to crosslink upon exiting the coaxial nozzle.

### Gene Delivery in Cell-Laden Ultrasound-Responsive Alginate Bioinks

Once the cell-laden ultrasound-responsive constructs had been printed, 400 µL of respective complete media was added to each of the bioprinted constructs and they were placed into an incubator at 37 °C, 5% CO_2_ for 15 min to dissolve gelatin on the outside of the constructs. The media was changed once to completely remove the excess gelatin. The media was removed from the bioprints before continuing.

To facilitate the use of focused ultrasound, 250 µL of 2.7 mg/mL type I collagen solution (Collagen from rat tail tendon, Roche) in acetic acid that had been pH neutralized with NaOH to an isotonic solution was pipetted on top of each construct. The dish was then rocked gently to ensure even distribution of the collagen. Next, the dish was placed into an incubator at 37 °C, 5% CO_2_ for 45 min, polymerizing the collagen. Upon collagen casting, the bioprinted filaments were completely embedded in the gelled collagen, which facilitated the use of focused ultrasound by holding the filament in place and minimizing interface density differences that can scatter the focused ultrasound beam. After the collagen had gelled, 400 µL of the cell-appropriate complete media was added to each construct-containing well.

The bioprinted constructs were then loaded into a custom ultrasound/optical microscope setup consisting of a 2.25 MHz spherically-focused ultrasound transducer (Olympus V305-SU) mounted in a tank of DI water, a robotic arm to position the sample, and a Nikon microscope body with high-speed camera (Photometrics Prime 95B sCMOS) attached to a 4X air objective lens (Nikon). The ultrasound focal zone was colocalized with the optical focal zone of the microscope objective. The ultrasound transducer was driven by a Class AB ultrasonic amplifier (Vox Technologies) and an arbitrary waveform generator (National Instruments PCI-5412). LabView 2016 software (National Instruments) was used to integrate the transducer, camera, and robotic arm for sample movement. Next, the ultrasound was focused 250 µm deep into the construct and a 10 ms focused ultrasound pulse (2.25 MHz, approximately 1.8 MPa peak negative pressure) was applied for 10, 40, or 80 pulses at 1 second intervals. The constructs were then placed back into the incubator at 37 °C, 5% CO_2_ for 48 hours before assessing gene expression with a media change at 24 hours.

### Fixing and Staining Cell-Laden Constructs for Gene Delivery Visualization

48 hr after ultrasound exposure, cell-laden constructs were fixed for 45 min at room temperature with 4% paraformaldehyde (Pierce™ 16% Formaldehyde (w/v), Methanol-free, Thermo Scientific) in + Ca/+ Mg 1X DPBS (Gibco). Each sample was rinsed three times with 400 µL of + Ca/+ Mg 1X DPBS. Samples were then permeabilized for 30 min at room temperature with 0.2% Triton X-100 (Thermo Scientific) in + Ca/+ Mg 1X DPBS. The solution was removed from the constructs and 32.4 µM Hoechst nuclear stain (Hoechst 33342, Trihydrochloride, Trihydrate, Invitrogen) in 0.2% Triton X-100, + Ca/+ Mg 1X DPBS was added to each construct for 1 hour at room temperature. After staining, each sample was washed 3 times for 15 minutes in the incubator at 37 °C, 5% CO_2_ with + Ca/+ Mg 1X DPBS before imaging.

### Brightfield and Fluorescence Microscopy and Image Analysis

Fluorescence microscopy was performed using the Leica Thunder 3D cell culture widefield microscope imaging system. Samples were imaged in brightfield and fluorescence with three replicate samples per condition. Each sample was imaged while maintaining the same image acquisition settings including exposure time and laser intensity across all samples within experiments. Image analysis for quantification of the microbubble activation zone within filaments and microbubble stability were performed using ImageJ as described in the sections below. Prior to measurements and analysis, the image size scale was calibrated in ImageJ using image scale information from the calibrated microscope. The analyses of transfection and cell viability were performed using Volocity Image Processing Software.

### Analysis of Bioprinted Microbubble Stability Over Time

To determine the stability of bioprinted microbubbles over time, filaments of 4% w/v sodium alginate containing HEK293T cells and 2.34 × 10^9^ µB/mL were bioprinted as previously described. Immediately post-printing, the constructs were cast in collagen and allowed to crosslink in the incubator as described above. After adding 400 µL of complete media to each well, dishes containing the filaments were sparged with octofluoropropane gas and parafilmed to promote stability. Room temperature (RT) samples were left on the benchtop, while incubated samples (37 °C) were placed into the incubator. Each timepoint and temperature condition had 3 replicates. At each timepoint, (0 hr, 1 hr, 3 hr, 6 hr, 12 hr, 24 hr, 36 hr, and 48 hr post-printing) samples were imaged using brightfield microscopy on the Leica Thunder widefield microscope. Microbubble stability was determined by measuring transmitted light through the filament at set time points. If microbubbles destabilized, they would no longer scatter transmitted light and more transmitted light would pass through the filament. To assess stability by image analysis, using ImageJ, a circular ROI 500 µm in diameter was placed within the filaments, and the average pixel intensity was calculated and averaged among 3 samples for each condition. The baseline of 100% intensity was established for samples at 0 hr, to which all other samples were compared. For each timepoint, normalization was done by dividing the average 0 hr pixel intensity by the average pixel intensity of that timepoint and multiplying by 100.

### Analyzing Size of the Visual Activation Zone of Microbubbles Post-Ultrasound

Bioinks consisting of 4% w/v sodium alginate containing 1.56 × 10^9^ µB/mL, 2.34 × 10^9^ µB/mL, and 3.51 × 10^9^ µB/mL were prepared, bioprinted, and prepared for ultrasound exposure as previously described (n = 3 replicate samples per condition). All samples were then imaged using brightfield microscopy on the Leica Thunder widefield microscope. After imaging, the filaments were individually exposed to 40 pulses of focused ultrasound, focused on the top plane of the filament, and imaged again post-ultrasound. Using ImageJ, a line, centered to the filament, was drawn along the length of the filament and a pixel intensity plot along the line was generated. Microbubbles scatter transmitted light, therefore regions containing more microbubbles have lower pixel intensities than regions with fewer microbubbles. The pixel intensity for the top 200 µm of the line plot was averaged to define a baseline and compared to intensity values further down the filament. The top edge of the ultrasound activation zone was defined as where the value on the line profile exceeded three times the average baseline intensity value. This analysis was repeated for the bottom edge of the activation zone using the bottom 200 µm of the line plot to define the lower baseline value. The distance between the top and bottom edges was measured and reported as the diameter of the activation zone.

### Analysis of Cell Viability in Alginate Bioprinted Constructs

Bioinks of 2%, 4%, and 6% w/v alginate containing HEK293T cells and 2.34 × 10^9^ µB/mL were prepared and printed as previously described. At either 0 hr post-printing or 48 hr post-printing, the filaments were stained with calcein-AM (LIVE/DEAD™ Viability/Cytotoxicity Kit, for mammalian cells, 8 µM, ThermoFisher) and ethidium homodimer-1 (LIVE/DEAD™ Viability/Cytotoxicity Kit, for mammalian cells, 4 µM, ThermoFisher) diluted in complete media (DMEM, 10% FBS, 1% pen-strep) for 45 min in the incubator. Samples stained at 48 hr post-printing were given media changes daily prior to staining. After staining as aforementioned, the filaments were washed with complete media three times for 5 min each in the incubator. The filaments were then imaged using fluorescence microscopy on a Leica Thunder widefield microscope. To determine viability, Volocity Image Processing software was used to segment and count the number of live and dead cells within each filament. Viability was calculated by dividing the number of live cells by the number of total cells (live + dead), then multiplying by 100 to obtain a percentage. Each condition had three replicate samples.

### Analysis of Ultrasound Pulse Number Effect on Cell Transfection

Bioinks containing 4% w/v alginate with HEK293T cells and 2.34 × 10^9^ µB/mL with coupled GFP plasmid were bioprinted and prepared for ultrasound exposure as previously described. Samples were imaged using brightfield microscopy before ultrasound exposure. Each sample was then exposed to 10, 40, or 80 ultrasound pulses focused at a depth of 250 µm into the filament (n = 3 replicate samples per condition) and imaged again in brightfield. The samples were then placed back into the incubator with daily media changes until the 48 hr post-ultrasound timepoint. Control samples exposed to 0 ultrasound pulses (n = 3 replicate samples) were prepared with the same protocol. All samples were fixed and stained with Hoechst nuclear stain as previously described before imaging using brightfield and fluorescence microscopy on the Leica Thunder widefield microscope. Volocity Image processing software was used to segment and count the number of GFP transfected cells in each filament. To determine the diameter of the zone containing transfected cells, ImageJ was used to draw a straight vertical line parallel to the two transfected cells furthest from each other in each bioprinted sample. This line was then measured and reported as the diameter of the zone containing transfected cells.

### Analysis of Microbubble Concentration Effect on Transfected Cells

Bioinks containing 4% w/v sodium alginate with HEK293T cells and 7.81 × 10^8^ µB/mL, 1.56 × 10^9^ µB/mL, 2.34 × 10^9^ µB/mL, and 3.51 × 10^9^ µB/mL were prepared, bioprinted, and prepared for ultrasound exposure as previously described. All samples were then imaged before ultrasound using brightfield microscopy. Each sample was then exposed 40 ultrasound pulses focused at a depth of 250 µm into the filament (n = 3 replicate samples per condition) and imaged again in brightfield. The samples were then placed back into the incubator with daily media changes until the 48 hr post-ultrasound timepoint. Control samples containing 2.34x10^9^ µB/mL with no plasmid DNA, and containing no microbubbles and no DNA were prepared and exposed to ultrasound with the same protocol (n = 3 replicate samples per condition). All samples were fixed and stained with Hoechst nuclear stain as previously described before imaging using brightfield and fluorescence microscopy on the Leica Thunder widefield microscope. Volocity Image processing software was used to segment and count the number of GFP transfected cells in each filament. To determine the diameter of the zone containing transfected cells, ImageJ was used to draw a straight vertical line parallel to the two transfected cells furthest from each other in each bioprint as described in the previous section.

### Statistical Analysis

All quantitative experiments were performed with a minimum of n = 3 replicates. Unless otherwise noted, the data presented are means +/− standard deviation. All statistical analyses were performed using GraphPad Prism 10 Software. One-way analysis of variance (ANOVA) with Tukey’s *post hoc* multiple comparisons tests were performed on each measurement with multiple groups compared.* p* values of < 0.05 were considered significant and denoted with * (*p < 0.05, **p < 0.01, ***p < 0.001, ****p < 0.0001).

## Results

### Coupling Ultrasound-Responsive Microbubbles to Plasmid DNA

**Fig. 2:**
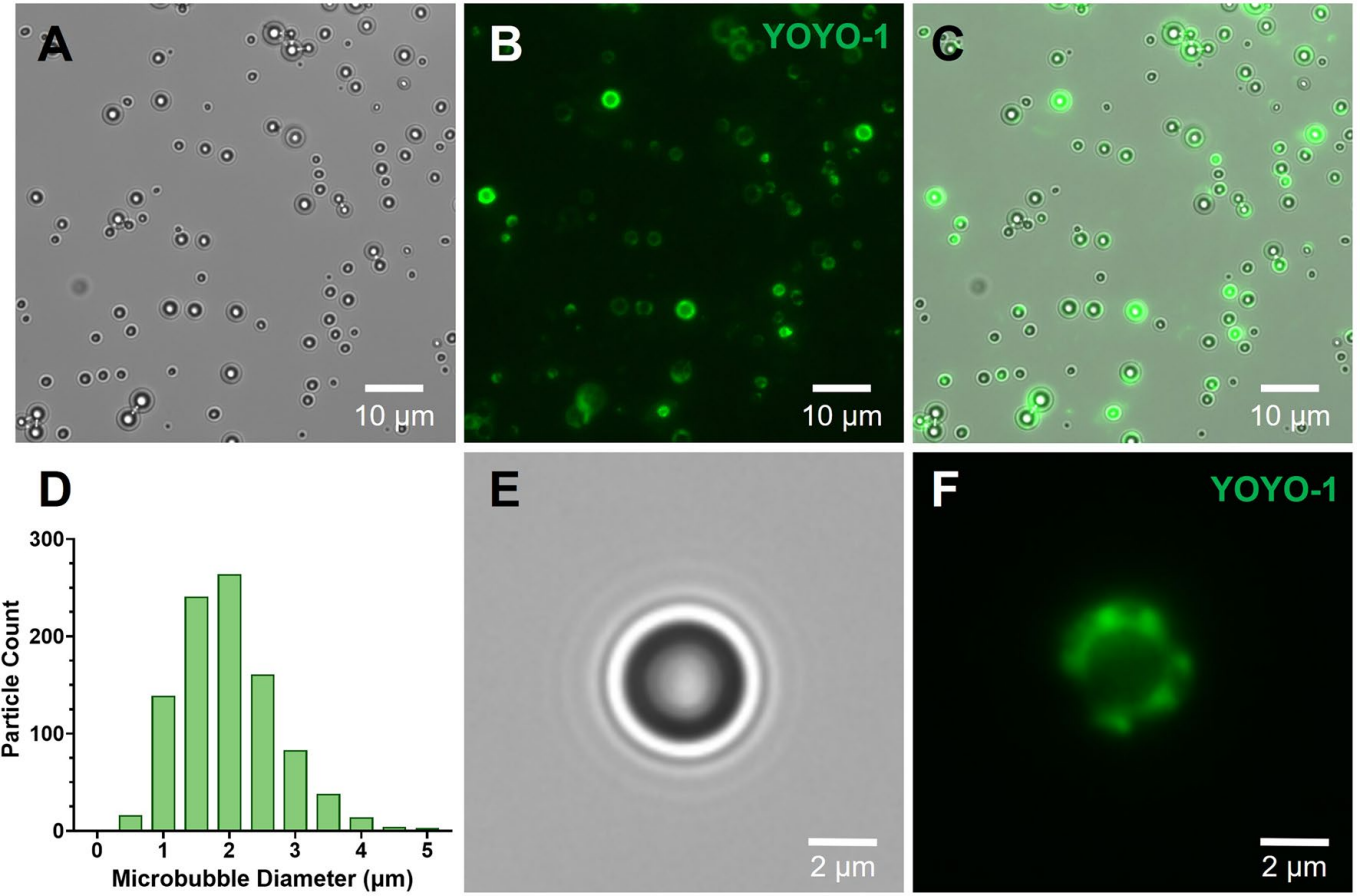
Ultrasound-responsive microbubble gene delivery vehicles. **A-C** Representative microscopy images of DNA-coupled ultrasound-responsive microbubbles in **A** brightfield, **B** fluorescence (green; plasmid DNA visualized via YOYO-1 stain), and **C** brightfield/fluorescence overlay (green; plasmid DNA visualized via YOYO-1 stain). **D** Representative size distribution of DNA-coupled ultrasound-responsive microbubbles. **E-F** Enlarged microscopy image of DNA-coupled ultrasound-responsive microbubble in **E** brightfield, showing the dark microbubble and characteristic ring distortions surrounding it due to the lensing effect of the gas bubble core, and **F** fluorescence (green; plasmid DNA visualized via YOYO-1 stain)

We developed a method for coaxially bioprinting DNA-coupled ultrasound-responsive microbubbles in cell-laden bioinks, where focused ultrasound was applied to these bioprinted constructs resulting in spatiotemporally controllable gene delivery (Fig. 1). To characterize the microbubble gene delivery vehicles, cationic lipid coated microbubbles were fabricated and electrostatically coupled to plasmid DNA, and the loaded DNA was visualized using YOYO-1 nucleic acid stain. Microbubbles were imaged using brightfield (Fig. 2A, E) and fluorescence microscopy (Fig. 2B, F). By overlaying the brightfield and fluorescence images, we observed that the fluorescent signal of the YOYO-1-stained DNA localized to the microbubbles (Fig. 2C) and showed stable DNA retention even after repeated washing. Microbubble size distribution was quantified by image analysis of the brightfield images, showing that the population of the microbubbles were between 1 and 5 µm in diameter (Fig. 2D).

### Coaxial Bioprinting of Ultrasound-Responsive Microbubbles in Bioinks of Increasing Alginate Concentrations

**Fig. 3:**
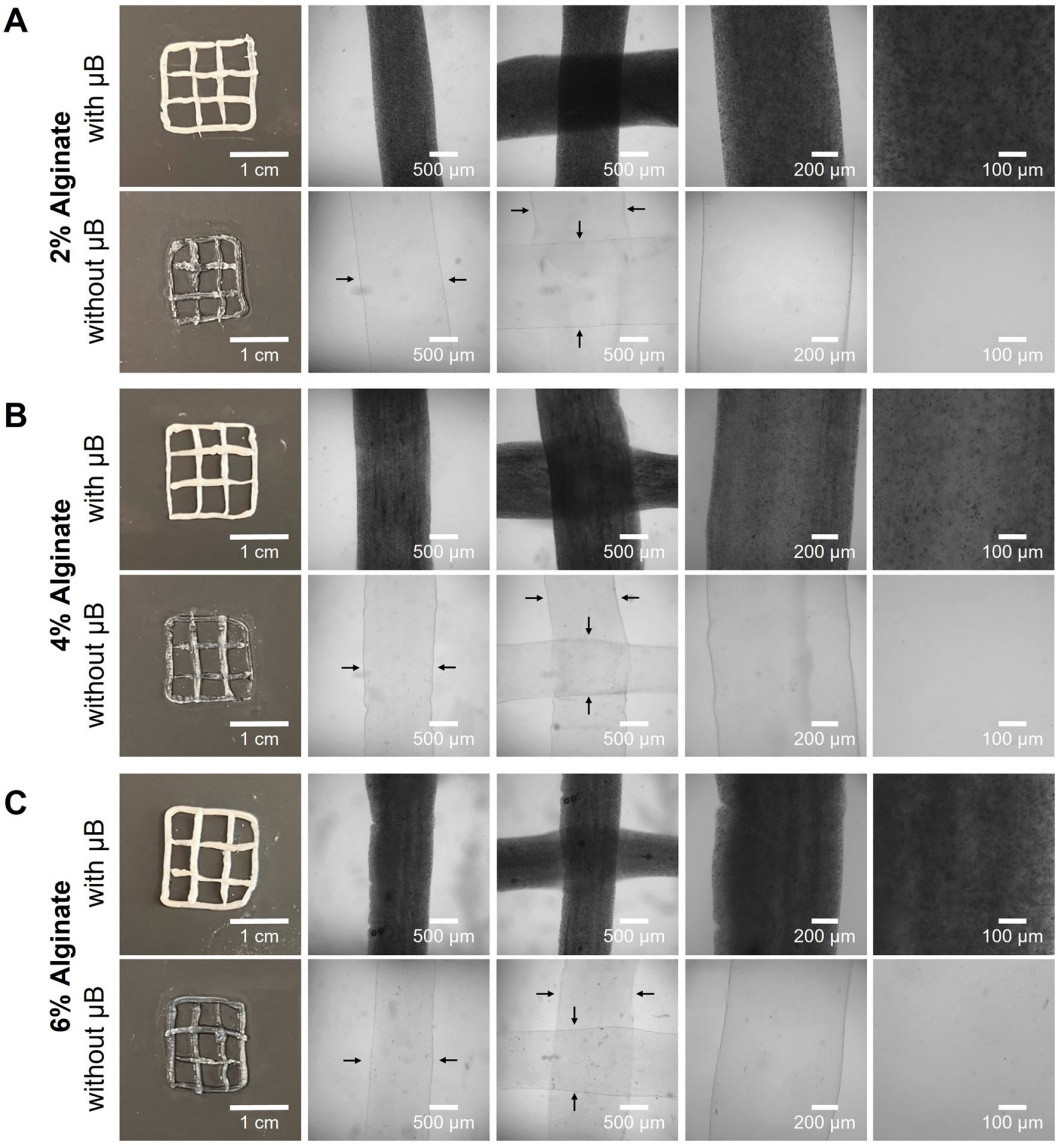
Coaxial bioprinting of microbubble gene delivery vehicles. **A**–**C** Representative macroscale and microscopy images of coaxially-bioprinted alginate at **A** 2% w/v, **B** 4% w/v, **C** 6% w/v, with and without ultrasound-responsive microbubble (µB) gene delivery vehicles. Black arrows indicate filament boundaries

We added microbubbles to varying concentrations of sodium alginate and coaxially bioprinted lattice constructs. Additionally, control constructs were printed without microbubbles for comparison. The 2% alginate (Fig. 3A) required a printing pressure of 10–12 kPa and was the most challenging bioink to print due to its low viscosity making it easy to over-extrude. The 4% alginate (Fig. 3B) required a printing pressure of 11–13 kPa and was the easiest to print and maintain print fidelity. Finally, the 6% alginate (Fig. 3C) required a printing pressure of 13–15 kPa and was similar to the 4% alginate in print ease and fidelity. Bioprinted constructs were imaged using a digital camera, to capture the entire construct, as well as brightfield microscopy.

The bioinks behaved similarly with and without microbubbles. At all alginate concentrations, the presence of the microbubbles did not cause any observable differences in printability or required extrusion pressure. The incorporated microbubbles are visible in the bioprinted filaments in the widefield macroscale views as the opaque white coloration. When viewed with trans-illumination brightfield microscopy, the microbubbles scattered the light and caused the filaments to appear dark. When viewed at high magnification, individual microbubbles are visible as dark dots within the filaments. The filaments appear clear and without coloration when microbubbles are not present. As supported by the macroscale and microscopy filament images (Fig. 3), the microbubbles maintained stability throughout the printing and imaging process.

### Stability of Ultrasound-Responsive Microubbles in Bioprinted 4% w/v Alginate Constructs

**Fig. 4:**
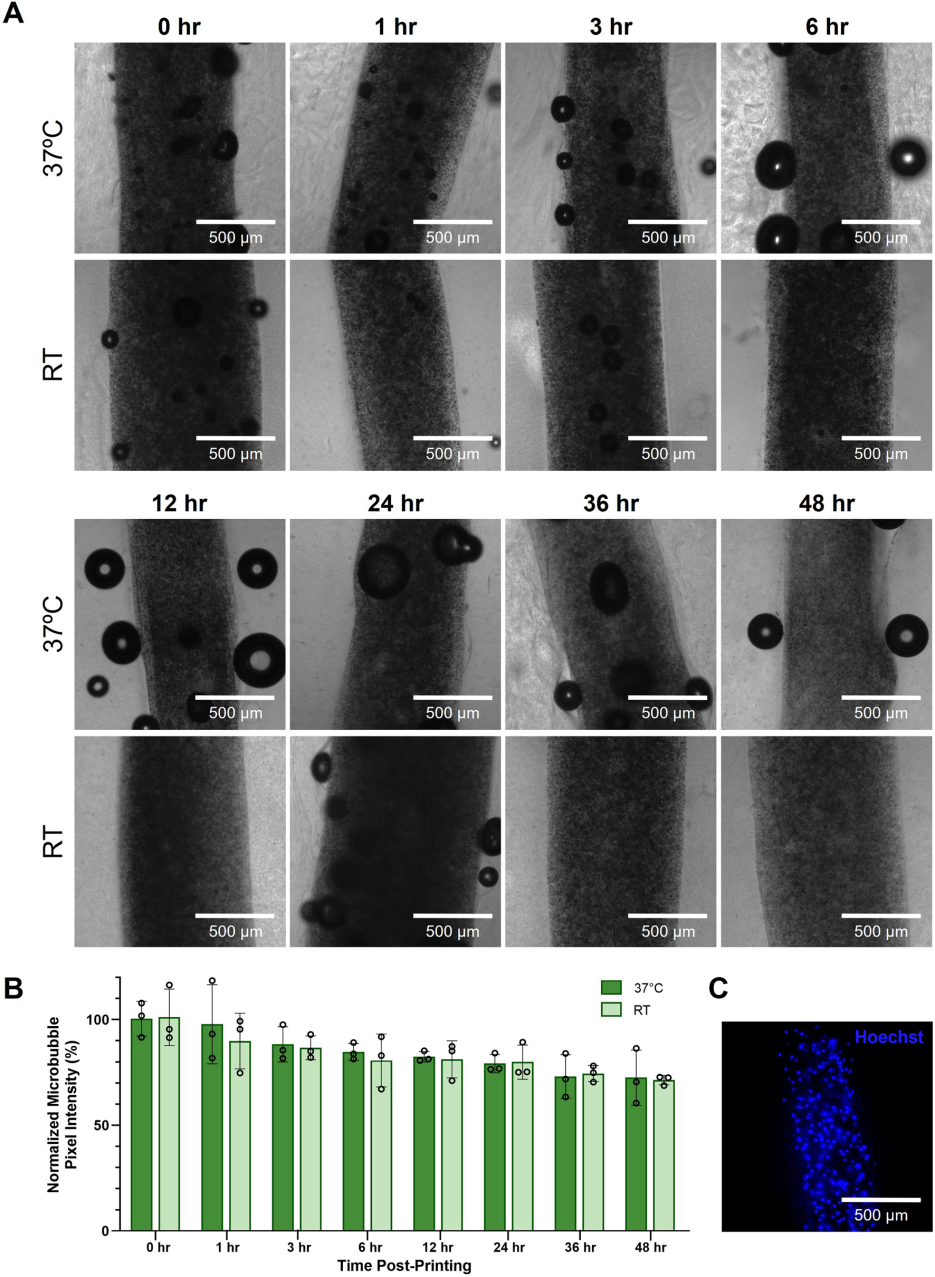
Stability of coaxially-printed microbubble gene delivery vehicles. **A** Representative microscopy images of coaxially-bioprinted HEK293T-laden 4% w/v alginate filaments containing microbubbles from 0-48 hr, in samples kept incubated at 37 °C (top row) and at room temperature (RT, bottom row). **B** Quantification of microbubble stability over time in 4% w/v alginate in samples incubated at 37 °C and at room temperature. **C** Representative fluorescence image of bioprinted filament containing microbubbles and Hoechst stained HEK293T cells

After demonstrating that we were able to stably print the alginate bioinks with incorporated ultrasound-responsive microbubbles, we sought to determine how long the microbubbles remained stable in the printed constructs. Microbubbles were added to HEK293T-laden 4% w/v alginate, and filaments were coaxially bioprinted. The bioprints were placed either in the incubator at 37 °C, or at room temperature (RT). Immediately post-printing (0 hr), the bioprints were imaged to determine the starting pixel intensity of transmitted light through the filaments. Samples were then imaged at various timepoints over 48 hr to determine how long the microbubbles remained stable (Fig. 4). At 48 hrs, it was observed that over 70% of the microbubble scattering signal was still present.

### Ultrasound Activation of Microbubbles in 4% w/v Alginate Bioprinted Constructs

**Fig. 5:**
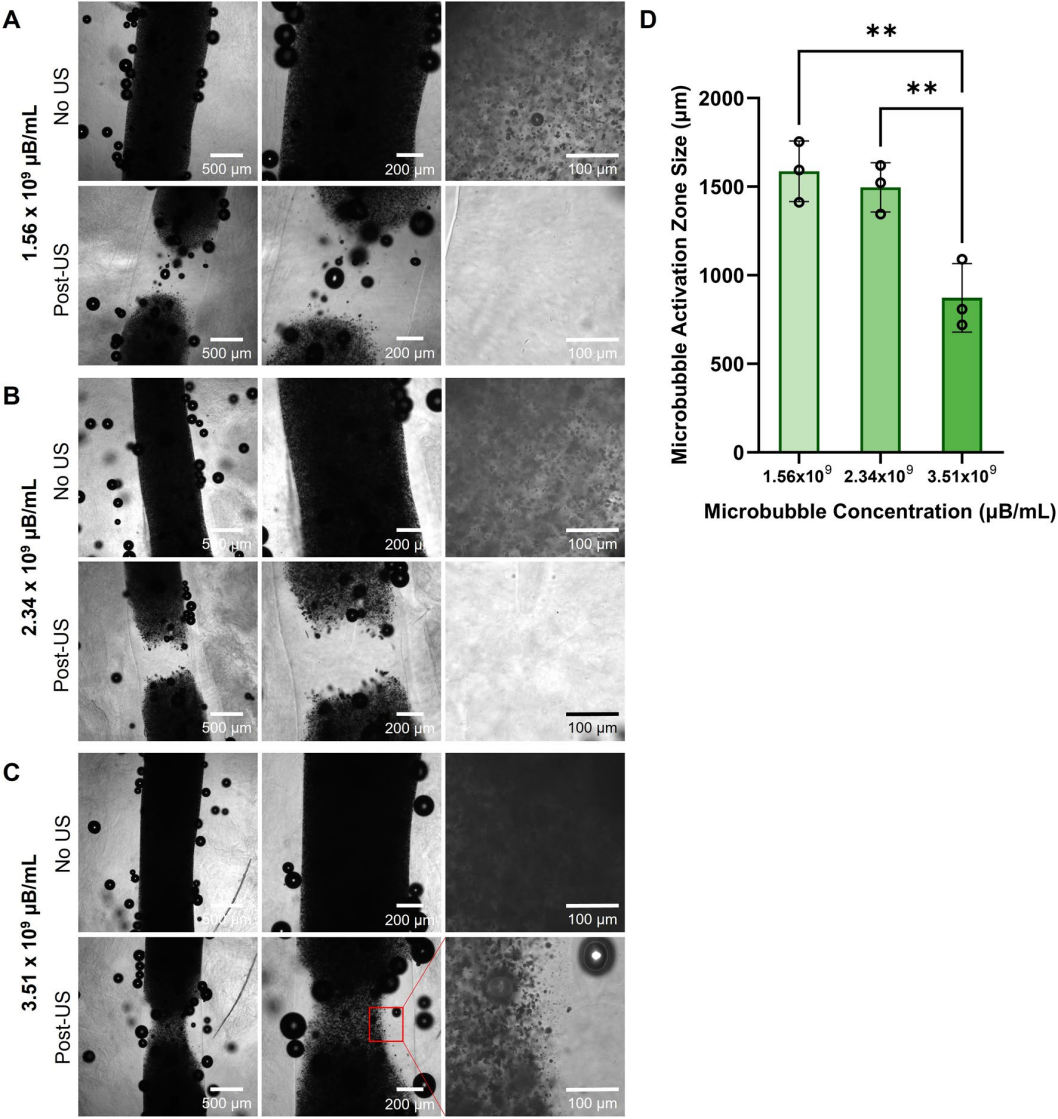
Effect of microbubble concentration on the size of the ultrasound induced activation zone in 4% w/v alginate bioprinted constructs. **A–C** Brightfield images of 4% w/v alginate pre- and post-ultrasound (US) exposure containing **A** 1.56 × 10^9^ µB/mL **B** 2.34 × 10^9^ µB/mL and **C** 3.51 × 10^9^ µB/mL. **D** Quantified size of microbubble activation zone in 4% alginate with varying microbubble concentrations (**p < 0.01, n = 3, one-way ANOVA, Tukey’s multiple comparisons test, error bars denote standard deviation)

To investigate the effect that bioink microbubble concentration had on the ultrasound-induced region of microbubble activation, we varied the concentration of microbubbles in 4% w/v alginate (Fig. 5A–C, top row) and imaged using brightfield microscopy before exposing each filament to 40 pulses of focused ultrasound. The prints were imaged again post-ultrasound exposure (Fig. 5A–C, bottom row), and the size of the microbubble activation zone was assessed by image analysis (Fig. 5D). Overall, the filaments with the two lower microbubble concentrations had larger activation zone sizes of 1587 ± 171 µm and 1496 ± 139 µm, respectively. The filaments with the highest microbubble concentration had a significantly smaller activation zone size of 873 ± 194 µm with incomplete ultrasound activation of the microbubbles in the focal zone. This supports an inverse trend between the microbubble concentration and the focal zone size; as the concentration of microbubbles increased, the size of the microbubble activation zone decreased.

### Viability of Cells in Bioprints with Ultrasound-Responsive Microbubbles and Increasing Alginate Concentration

**Fig. 6:**
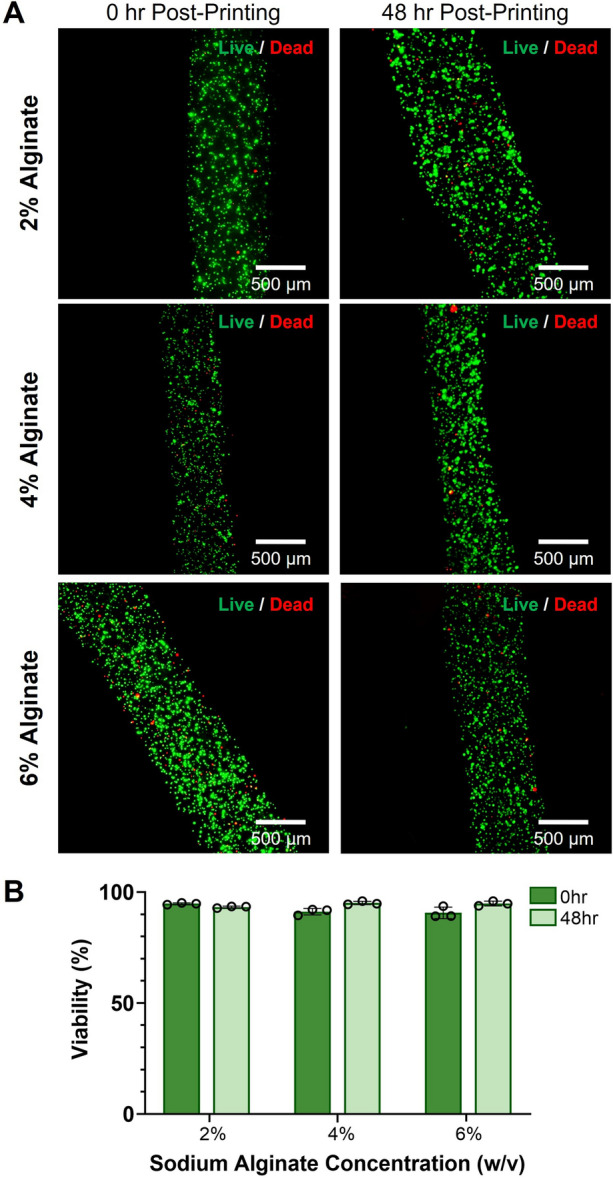
Viability of bioprinted cells with ultrasound-responsive microbubbles in alginate bioinks. **A** Representative fluorescence microscopy images of 2%, 4%, and 6% w/v alginate bioprints containing HEK293T cells and microbubbles at 0 hr and 48 hr post-printing. Constructs were stained with calcein-AM (green, live cells) and ethidium homodimer-1 (red, dead cells).** B** Viability of cells printed in 2%, 4%, and 6% w/v alginate at 0 hr and 48 hr post-printing (n = 3, error bars denote standard deviation)

To ensure that we could bioprint cells with microbubbles and maintain high cell viability at different alginate concentrations, we printed HEK293T cells in varying w/v sodium alginate containing microbubbles. We stained bioprinted constructs 0 hr and 48 hr post printing, as shown by representative filaments in Fig. 6A. The cells in the bioprints maintained a high viability of over 85% in all alginate concentrations from 0 to 48 hr post-printing (Fig. 6B).

### Effect of Varying the Number of Ultrasound Pulses on Ultrasound-Controlled Cellular Transfection in Coaxially-Bioprinted Constructs

**Fig. 7:**
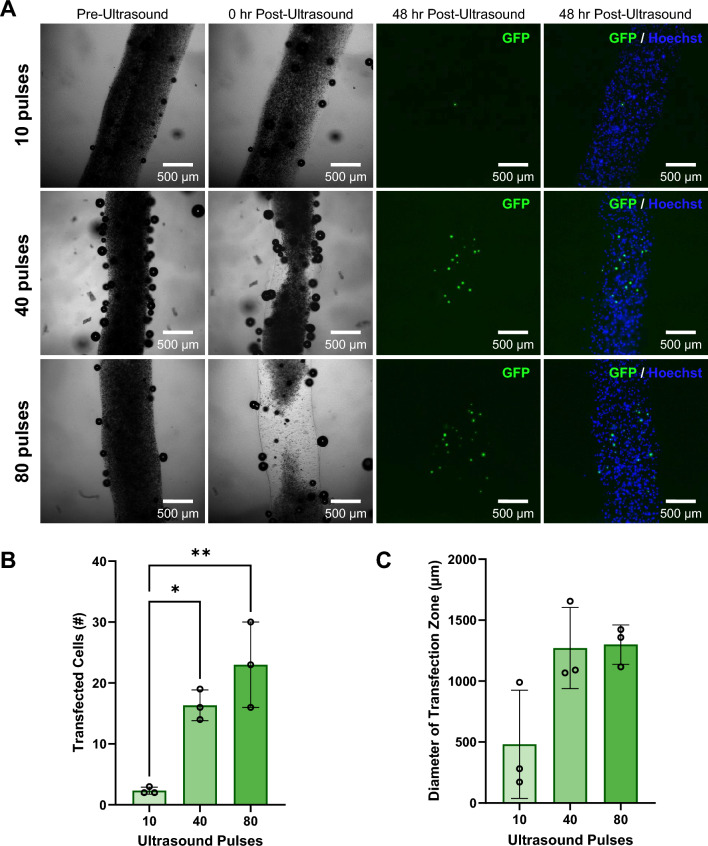
Effect of varying the number of ultrasound pulses on ultrasound-controlled transfection of HEK293T cells in coaxially-bioprinted constructs. **A** Coaxially-bioprinted HEK293T-laden 4% alginate bioink containing GFP-coupled microbubbles before ultrasound, 0 hr post-ultrasound, and 48 hr post-ultrasound with varying ultrasound exposure (10, 40, or 80 pulses). **B** Number of transfected cells in bioprinted constructs at 48 hr post-ultrasound for varying numbers of ultrasound pulses (*p < 0.05, **p < 0.01, one-way ANOVA, Tukey’s multiple comparisons test, error bars denote standard deviation). **C** Diameter of zone containing transfected cells at 48 hr post-ultrasound for varying numbers of ultrasound pulses (one-way ANOVA, Tukey’s multiple comparisons test, error bars denote standard deviation)

The number of focused ultrasound pulses that a construct is exposed to is a parameter that affects microbubble activation, which in turn affects the number of transfected cells in the ultrasound focal zone. To investigate the ability to modulate transfection with ultrasound exposure, we exposed bioprinted 4% alginate filaments containing GFP plasmid microbubbles and HEK293T cells to varying numbers of focused ultrasound pulses and imaged pre- and post-ultrasound to visualize the region of microbubble activation. After 48 hours, the samples were fixed and stained with Hoechst nuclear stain and analyzed for GFP expression by fluorescence microscopy (Fig. 7A). The number of transfected cells in each construct ranged from 2-30 cells per construct, with the fewest pulses (10) having an average of 2 transfected cells. The constructs exposed to 40 pulses had an average of 16 transfected cells, and the constructs exposed to 80 pulses had an average of 23 transfected cells (Fig. 7B). We observed that as the number of ultrasound pulses increased, there was an increasing trend in the number of transfected cells within the bioprinted constructs. Upon measuring the diameter of the zone containing transfected cells along the filaments (the effective transfection zone diameter), there was no significant change observed in effective transfection zone diameter among the groups exposed to different numbers of ultrasound pulses (Fig. 7C). Control samples that were not exposed to ultrasound pulses did not show any evidence of transfection when imaged and analyzed at 48 hr, indicating that ultrasound stimulus application is necessary for gene delivery (Fig. S1). In all ultrasound-exposed samples, the boundaries of the filaments remained visible and uniform in appearance at the 48 hr time point.

### Effect of Varying Microbubble Concentration on Ultrasound-Controlled Cellular Transfection in Coaxially-Bioprinted Constructs

**Fig. 8:**
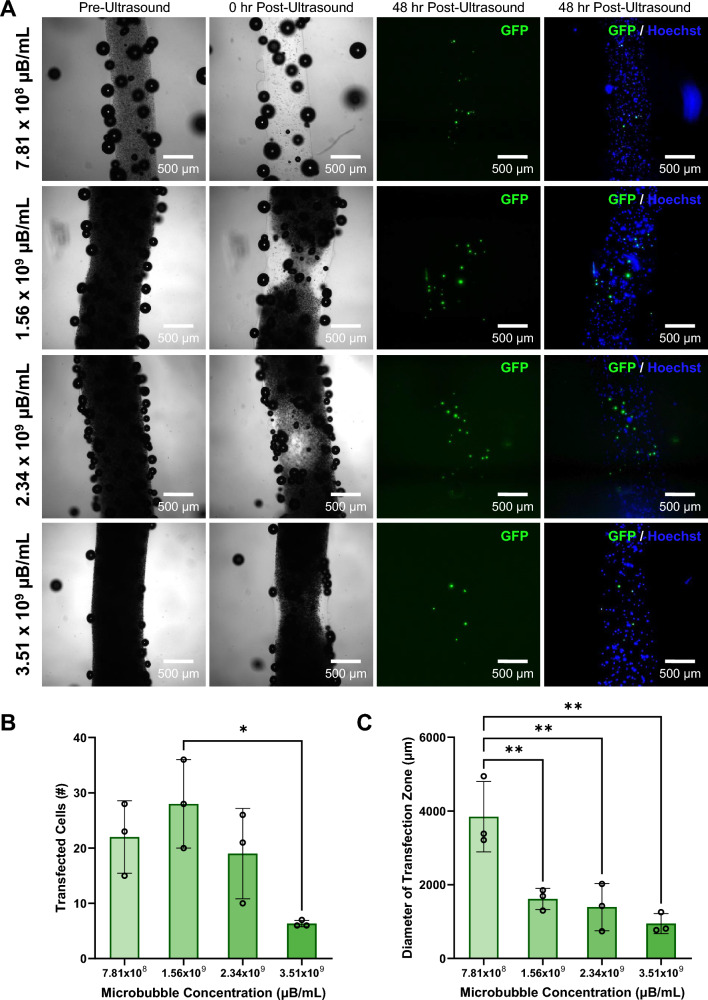
Effect of increasing microbubble concentration on ultrasound-controlled HEK293T cellular transfection in coaxially-bioprinted constructs. **A** Increasing concentrations of GFP-coupled microbubbles in coaxially-bioprinted HEK293T-laden 4% alginate bioink, before ultrasound, 0 hr post-ultrasound, and 48 hr post-ultrasound. **B** Number of transfected cells in bioprinted constructs at 48 hr post-ultrasound for varying microbubble concentrations (*p < 0.05, one-way ANOVA, Tukey’s multiple comparisons test, error bars denote standard deviation). **C** Diameter of zone containing transfected cells at 48 hr post-ultrasound for varying microbubble concentrations (**p < 0.01, one-way ANOVA, Tukey’s multiple comparisons test, error bars denote standard deviation)

To determine the effect of microbubble concentration within the cell-laden bioprints on the number of ultrasound transfected cells and the size of the zone in which cells are transfected, we added GFP-plasmid loaded microbubbles at multiple concentrations to 4% alginate containing HEK293T cells and coaxially bioprinted filaments as previously described, imaging the filaments before and immediately after ultrasound exposure. After 48 hours, the samples were analyzed for GFP expression by fixing and staining with Hoechst nuclear stain and then imaged with fluorescence microscopy (Fig. 8A).

Microbubble concentration was observed to affect the number of transfected cells, with the highest average transfection occurring at the intermediate concentration of 1.56 × 10^9^ µB/mL. Transfection was observed to decrease as microbubble concentration increased above this (Fig. 8B). The lowest microbubble concentration was observed to have the largest diameter zone of transfected cells (Fig. S4), and increasing microbubble concentration showed a decrease in the diameter of the transfection zone (Fig. 8C). Control samples containing no microbubbles that were exposed to ultrasound showed no transfected cells. This indicates the presence of gene delivery microbubbles is necessary for DNA delivery (Fig. S2). Additional control samples containing microbubbles with no GFP-plasmid were treated with ultrasound exposure, and no transfected cells were observed (Fig. S3).

### Modulation of Osteoblast Transfection in Coaxially-Bioprinted Constructs

**Fig. 9:**
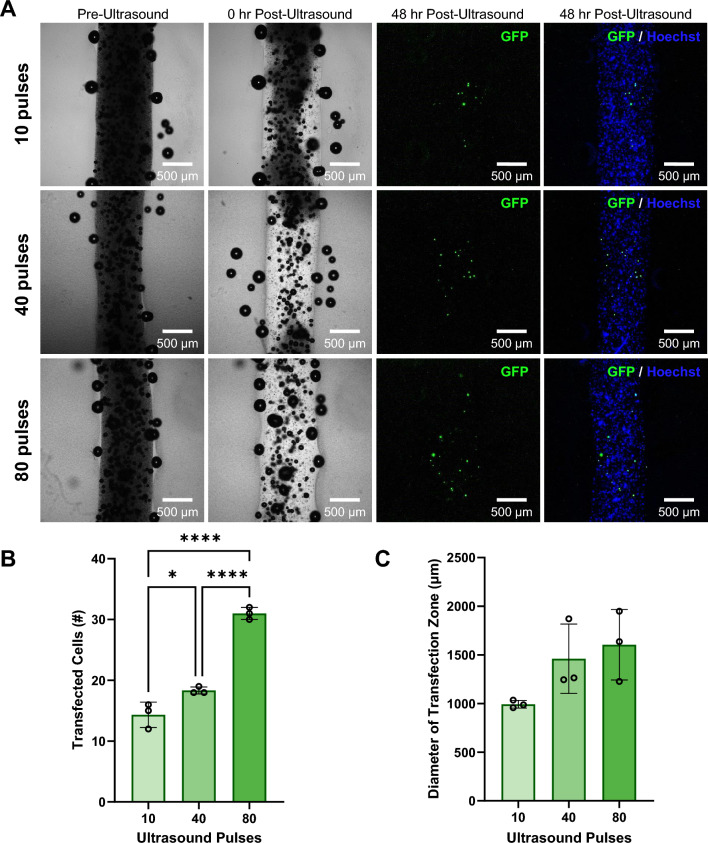
Effect of varying the number of ultrasound pulses on ultrasound-controlled osteoblast cellular transfection in coaxially-bioprinted constructs. **A** Coaxially-bioprinted osteoblast-laden (hFOB 1.19) 4% alginate bioink containing GFP-coupled microbubbles before ultrasound, 0 hr post-ultrasound, and 48 hr post-ultrasound with varying ultrasound exposure (10, 40, or 80 pulses). **B** Number of transfected cells in bioprinted constructs at 48 hr post-ultrasound for varying numbers of ultrasound pulses (*p < 0.05, ****p < 0.0001, one-way ANOVA, Tukey’s multiple comparisons test, error bars denote standard deviation). **C** Diameter of zone containing transfected cells at 48 hr post-ultrasound for varying numbers of ultrasound pulses (one-way ANOVA, Tukey’s multiple comparisons test, error bars denote standard deviation)

Extending the technique of focused ultrasound-mediated genetic delivery in bioprints to additional cell types, we incorporated osteoblasts (hFOB 1.19) into our ultrasound-responsive bioinks and varied the number of ultrasound pulses applied (Fig. 9A). We observed that as the number of ultrasound pulses increased, there was an increasing trend in the number of transfected cells. The number of transfected cells in each construct ranged from 12-32 cells per construct, with the fewest pulses (10) having an average of 14 transfected cells (Fig. 9B). The constructs exposed to 40 pulses had an average of 18 transfected cells, and the constructs exposed to 80 pulses had an average of 31 transfected cells. There was no significant change observed in effective transfection zone diameter among the groups exposed to different numbers of ultrasound pulses (Fig. 9C). Control samples that were not exposed to ultrasound pulses did not show any evidence of transfection when imaged and analyzed at 48 hrs, indicating that ultrasound stimulus application is necessary for gene delivery (Fig. S5). Osteoblasts printed with no microbubbles and no plasmid also showed no visible transfection with applied ultrasound pulses (Fig. S6).

## Discussion

Here we describe the development of coaxially 3D bioprinted ultrasound-responsive scaffolds for remote-controlled gene delivery. This design uniquely enables ultrasound-mediated genetic manipulation of cells via microbubble gene delivery vehicles embedded within coaxially bioprinted cell-laden filaments. This ultrasound-mediated gene delivery technique has an advantage in 3D tissue scaffolds over traditional gene delivery methods that use vectors, such as viruses or lipofectamine, because the diffusion of these vectors is inhibited by the dense scaffold matrix making it a challenge to evenly deliver the DNA to cells in a controlled and predictable way. Diffusion mechanisms are also a challenge to localize to a desired region making it difficult for the user to target a particular region of cells to transfect. With diffusion, it is also difficult to control the timing of when the traditional vectors will reach a desired region of the hydrogel construct. The ultrasound-triggered microbubble delivery technique addresses these challenges by incorporating the DNA-loaded microbubbles in the liquid bioink achieving an even distribution across the whole bioprinted construct. The focused ultrasound trigger gives the user spatiotemporal control over when and where the cells are transfected.

We observed that higher concentrations of alginate used for microbubble-containing bioinks, such as 6% w/v, were more easily printable with the coaxial extrusion setup and maintained higher print fidelity than lower alginate concentrations, but even the 2% w/v alginate had readily achievable printability. These lower alginate concentrations were less viscous and required a lower extrusion printing pressure to maintain printability.

The inclusion of ultrasound-responsive microbubbles was found to have no apparent effect on print fidelity, printability, or required printing pressure of alginate bioink formulations. The microbubbles maintained particle integrity in multiple alginate concentrations and were stable throughout printing and imaging. High printing pressures could jeopardize microbubble stability and cause them to prematurely collapse during the printing process, so we ensured that our bioinks could be printed below 15 kPa for even the highest concentration of alginate, which is desirable for both microbubble stability and cell viability [60].

Our study of microbubble stability in bioprinted cell-laden alginate constructs demonstrates the ability of ultrasound-responsive microbubbles to maintain particle integrity and responsiveness over time in the constructs after the printing process is concluded. Over 70% of the microbubble scattering signal was still present at 48 hr in both room temperature and 37 °C conditions. The 37 °C condition is valuable to support cell viability which enables user-defined temporal control over ultrasound-mediated gene delivery. This stability test was conducted at a microbubble concentration of 2.34 × 10^9^ µB/mL, and our results in Figure 8 show that a 1.5-fold and 3-fold reduction in microbubble concentration can achieve transfection in the print. Thus, it is possible that microbubbles could be loaded at higher concentrations in anticipation of the reduction in particles over time so that the correct number of microbubbles would be present at the desired time of activation. Future studies will characterize the ability to genetically manipulate bioprinted cells at multiple timepoints.

From our viability study, we observed that the bioprinting process had minimal adverse effects on cell viability. In all bioprinted samples, regardless of alginate bioink concentration, cell viability was over 85% at both 0 hr and 48 hr post-printing. This indicates that the pressure required to print our samples as well as time spent out of the incubator did not significantly affect cell viability. Additionally, our constructs maintained high cell viability for over 48 hr post-printing, which is a sufficient timepoint to assess our ultrasound-mediated cellular transfection. Our bioprinting method minimizes the time necessary for the cell-laden bioinks to remain outside of the incubator and affords the ability to maintain a set temperature of 37 °C during both the pre-printing and printing processes. Both the low printing pressures and minimized temperature variances could be factors attributing to the high viability of our bioprinted cells.

Microbubble concentration was found to inversely affect the size of the ultrasound activation zone of the microbubbles with lower concentrations of microbubbles having larger regions of activation. We also observed that microbubble concentration affects the number of transfected cells and the diameter of the transfection zone. The lowest concentration of microbubbles had the highest diameter region where the cells were transfected following focused ultrasound exposure. It has been reported that at high microbubble concentrations, the density of microbubbles can create a shielding effect reflecting some the ultrasound energy thereby shielding microbubbles from the full ultrasound intensity. Microbubbles closest to the transducer are the least shielded and collapse first [61]. As these microbubbles disappear, there is less of a shielding effect each time the construct is exposed to ultrasound [62]. This shielding effect explains our consistent observation that higher concentrations of microbubbles led to fewer microbubbles being activated via focused ultrasound, and therefore smaller microbubble activation zones compared to constructs with lower microbubble concentrations. Microbubble collapse dynamics can also be influenced by the distance between adjacent microbubbles [63] which changes with increasing concentrations loaded in the filament and could play a role in the activation differences observed.

Sonoporation is a physical process requiring no chemicals or active cellular processes, so it is a promising technique for genetic manipulation that can be applied to a wide range of cell types [45, 46]. We observed in both bioprinted HEK293T cells and bioprinted osteoblasts (hFOB 1.19) that as the number of focused ultrasound pulses applied to a bioprinted filament increased, the number of transfected cells increased, and the diameter of the zone of cell transfection increased. As more pulses of ultrasound are applied, fewer microbubbles remain in the sample to aid the shielding effect, which results in the activation of more microbubbles with each additional ultrasound pulse. This demonstrates that the number of transfected cells can be modulated by the number of focused ultrasound pulses applied to the bioprinted construct. The printed constructs containing osteoblasts had consistently higher numbers of transfected cells compared to the HEK293T cells, regardless of ultrasound pulse quantity. A potential explanation for this is that as the surface area of the cell increases, more microbubbles can interact with the cell surface, causing more transfection. Osteoblasts have a mean diameter of 25 µm [64], and HEK293T cells are smaller with a mean diameter of 15 µm [65]. It was observed that the diameter of the region containing transfected cells also had an increasing trend with the number of ultrasound pulses where we observed a ~ 2.5x increase in diameter from 10 to 80 pulses. It is worth noting that these transfection results are consistent with our observations that as microbubble concentration increased, the visually observed region of microbubble activation decreased, which is also likely due to the microbubble shielding effect. We showed robust control over the number of transfected cells in a bioprint, from about 2 to 30 cells, which demonstrates our ability to modulate the number of transgene-expressing cells, critical for studying diseases like cancer that initiate from mutations in a single cell or small cluster of cells [17, 66, 67].

Another important finding was that the number of transfected cells had an increasing trend as the microbubble concentration increased to 1.56 × 10^9^ µB/mL, but transfection decreased as microbubble concentration was further increased. A potential explanation for this is that at lower microbubble concentrations, there was less shielding and all of the ultrasound-responsive microbubbles in the construct were activated. As microbubble concentration started to increase, more bubbles were activated increasing the chances of successful cell transfection. As microbubble concentration increased further, it reached a point where the shielding effect actually reduced the number of activated microbubbles, thus reducing cellular transfection. This is consistent with the brightfield images showing that post-ultrasound, no microbubbles are seen to remain in the construct with the lowest starting microbubble concentration, but more microbubbles are present at the higher concentrations.

Together, these results indicate that there is a range of alginate concentrations, microbubble concentrations, and number of focused ultrasound pulses that are amenable to ultrasound-mediated genetic manipulation in our bioprinted constructs. Additionally, there is an optimal range of microbubble concentration and number of focused ultrasound pulses to control the number of manipulated cells and the diameter of the zone containing these transfected cells. We demonstrated a bioprinting method with the ability to modulate the aforementioned parameters to uniquely control the number of genetically manipulated cells, spatial aspects of the region containing these transfected cells, and the timepoint where genetic manipulation occurs. This is in contrast to standard methods of gene delivery in thick 3D constructs, which lack both spatial and temporal control due to challenges of vector diffusion.

This technology enables controlled genetic manipulation of cells in a variety of advanced biofabrication applications. The versatility of coaxial bioprinting allows the simultaneous and controlled deposition of multiple bioinks in the form of filaments, as we described here, and also hollow tube structures created by switching the sacrificial crosslinker bioink to the core of the coaxial needle. Additionally, concentric multi-material deposition enables the co-printing of bioinks that have desirable complimentary mechanical and biological properties, and the co-printing and controlled deposition of multiple cell types in a single construct [8]. Printing constructs that allow controlled genetic manipulation of incorporated epithelial, stromal, and immune cells [68, 69] would enable researchers to recapitulate important aspects of cellular interactions within the tissue microenvironment.

This technology will enable users to define when and where different genes can be overexpressed and can be used to study the role that genes of interest play in a variety of different biological processes including the maturation of tissue, wound healing, and disease progression. Targeted gene delivery could be used to spatially-control stimulated differentiation of stem cells as well as manipulate patient-derived cells to investigate disease progression and test drugs for patient-specific treatment options. A noteworthy potential application of this technology is the ability to manipulate a subset of established cells in a bioprinted microenvironment to overexpress oncogenes for modeling cancer progression in the 3D tissue context [17, 66, 70]. As sonoporation is a physical gene delivery method, as opposed to chemical or biological methods, this approach will likely be amenable to many cell types, including cells that are considered hard-to-transfect by chemical methods, as well as primary cells, stem cells, and non-immortalized cell lines. Additional optimization of ultrasound parameters such as the intensity or number of pulses applied may be required for these additional cell types. Future work will investigate different bioink formulations and the addition of multiple cell types to assess the effects of spatially and temporally controlled genetic manipulation on cellular interactions. This technology and future work will enable controlled genetic perturbation techniques to investigate cell crosstalk, migration, differentiation, and disease progression among other possibilities.

## Conclusions

We developed a new coaxial 3D bioprinting technique for ultrasound-controlled gene delivery in cell-laden bioprinted constructs. By incorporating ultrasound-responsive particles into extrudable bioinks and exposing the bioprinted constructs to focused ultrasound, we successfully demonstrated sonoporation of embedded cells, resulting in targeted DNA delivery and robust ultrasound-controlled transgene expression. Varying the concentration of ultrasound-responsive microbubble gene delivery vehicles and the number of applied focused ultrasound pulses allowed us to modulate the number of transfected cells and the region of the print where transgene was delivered in the bioprinted constructs. This platform enables remote-controlled genetic manipulation in coaxially bioprinted tissue constructs with the ability to spatiotemporally-define DNA delivery. This technology will enable future studies in tissue constructs coaxially printed with multiple bioinks and cell types. The ability to spatiotemporally-control genetic manipulation within coaxially bioprinted constructs can be leveraged in future studies to actuate intercellular communication, guide stem cell differentiation, and model disease states, with important applications in understanding disease progression and directing tissue regeneration.

## Supplementary Information

Below is the link to the electronic supplementary material.

Supplementary file1 (DOCX 1948 KB)

## Data Availability

The data that support the findings of this study are available from the corresponding author upon reasonable request.
