# Supplementary material for: Remote-Controlled Gene Delivery in Coaxial 3D-Bioprinted Constructs using Ultrasound-Responsive Bioinks
Source: Cell Mol Bioeng. 2024 Oct 27;17(5):401–21. doi: 10.1007/s12195-024-00818-x (PMC11538209; doi:10.1007/s12195-024-00818-x)
Supplement: Supplementary file 1 — Supplementary file1 (DOCX 1948 KB) [file 12195_2024_818_MOESM1_ESM.docx]

**Supplementary Information**

**Remote-Controlled Gene Delivery in Coaxial 3D-Bioprinted Constructs using Ultrasound-Responsive Bioinks**

Mary K. Lowrey^1,2^, Holly Day^1,2^, Kevin J. Schilling^1,2^, Katherine T. Huynh^1,2^, Cristiane M. Franca^2,3,4^, Carolyn E. Schutt^*1,2,3^

^1^ Biomedical Engineering Department, Oregon Health and Science University, Portland, OR 97201, USA

^2^ Cancer Early Detection Advanced Research Center, Knight Cancer Institute, Oregon Health and Science University, Portland, OR 97201, USA

^3^ Knight Cancer Precision Biofabrication Hub, Knight Cancer Institute, Oregon Health and Science University, Portland, OR 97201, USA

^4^ Department of Oral Rehabilitation and Biosciences, School of Dentistry, Oregon Health and Science University, Portland, OR 97201, USA

**
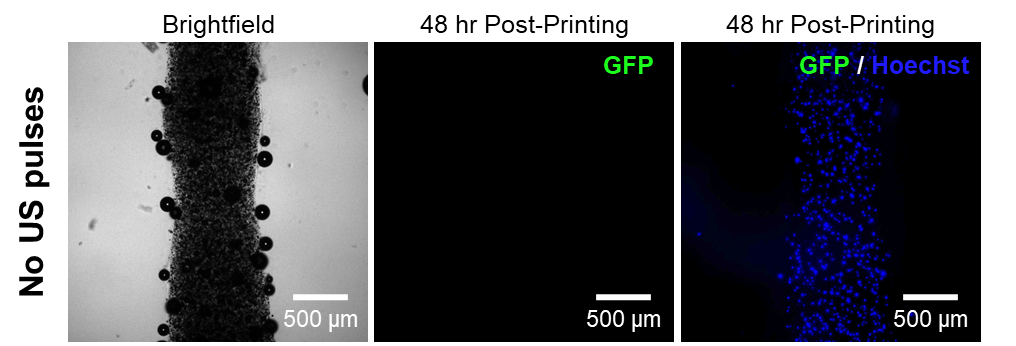
**

**Figure S1.** Representative images of coaxially-bioprinted HEK293T-laden 4% alginate bioink filament containing GFP-plasmid loaded microbubbles (at 2.34 x 10^9^ µB/mL) that was not exposed to ultrasound. Filament shown via brightfield microscopy (left), and fluorescence microscopy at 48 hr post-printing (middle, right).


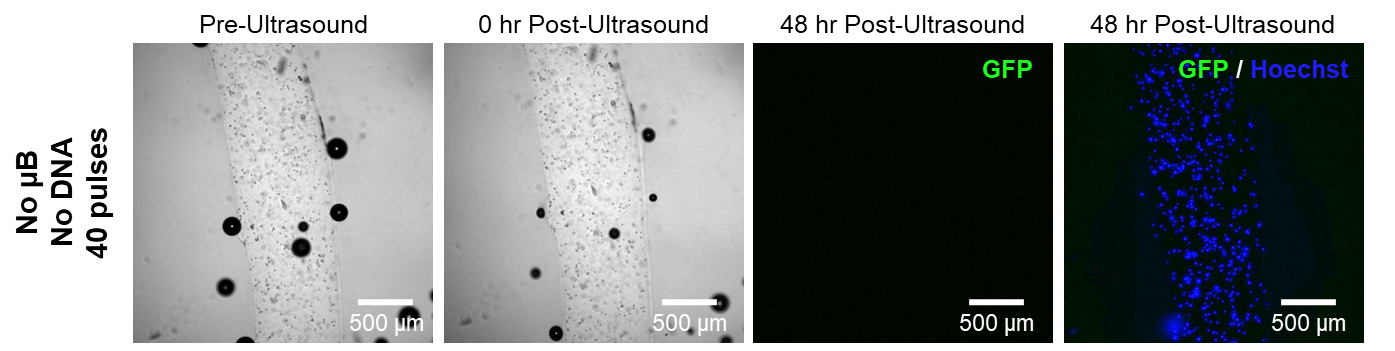


**Figure S2.** Representative images of coaxially-bioprinted HEK293T-laden 4% alginate bioink filament containing no microbubbles and no plasmid DNA, exposed to 40 ultrasound pulses. Filament shown in brightfield before and after ultrasound exposure, and via fluorescence microscopy at 48 hr post-ultrasound-exposure.


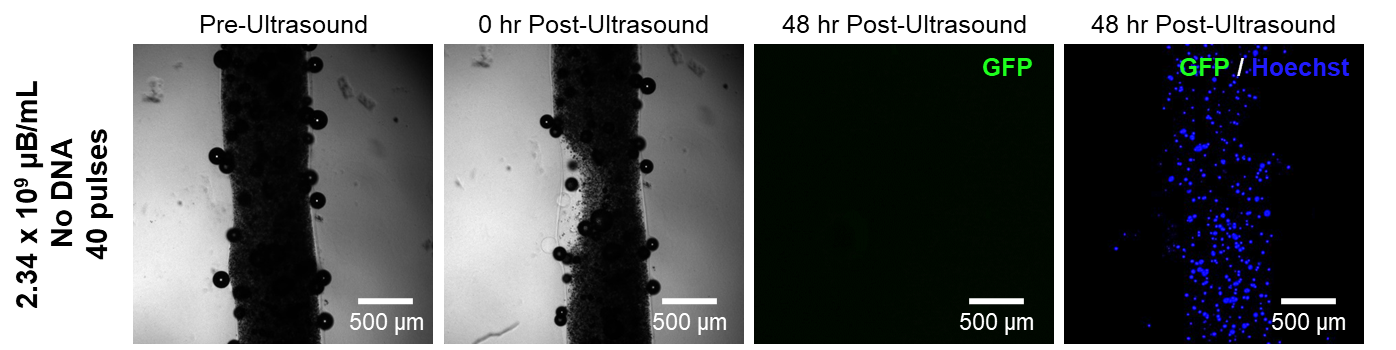


**Figure S3.** Representative images of coaxially-bioprinted HEK293T-laden 4% alginate bioink filament containing microbubbles with no plasmid DNA (at 2.34 x 10^9^ µB/mL), exposed to 40 ultrasound pulses. Filament shown in brightfield before and after ultrasound exposure, and via fluorescence microscopy at 48 hr post-ultrasound-exposure.


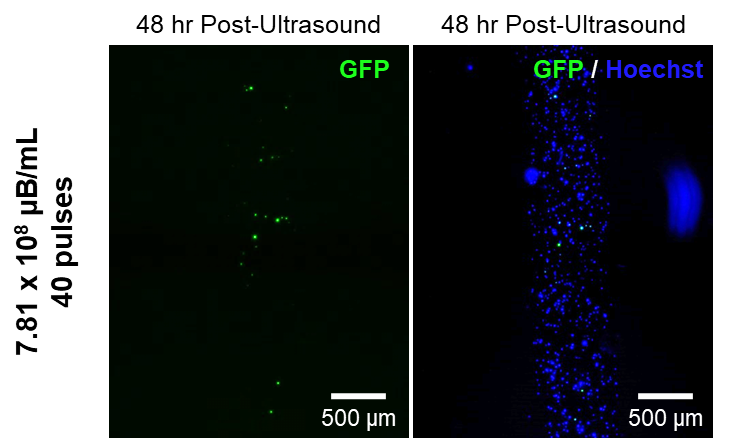


**Figure S4.** Expanded view of full transfection zone of coaxially-bioprinted HEK293T-laden 4% alginate filament printed with 7.81 x 10^8^ µB/mL and exposed to 40 ultrasound pulses. Expanded view obtained from stitched post-transfection fluorescence images from the sample appearing in Figure 8A.

**
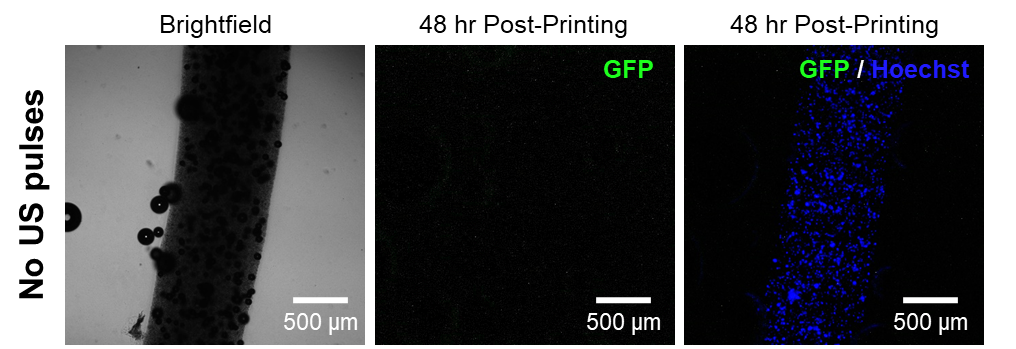
**

**Figure S5.** Representative images of coaxially-bioprinted osteoblast-laden (hFOB 1.19) 4% RGD-alginate bioink filament containing GFP-plasmid loaded microbubbles (at 2.34 x 10^9^ µB/mL) that was not exposed to ultrasound. Filament shown via brightfield microscopy (left), and fluorescence microscopy at 48 hr post-printing (middle, right).


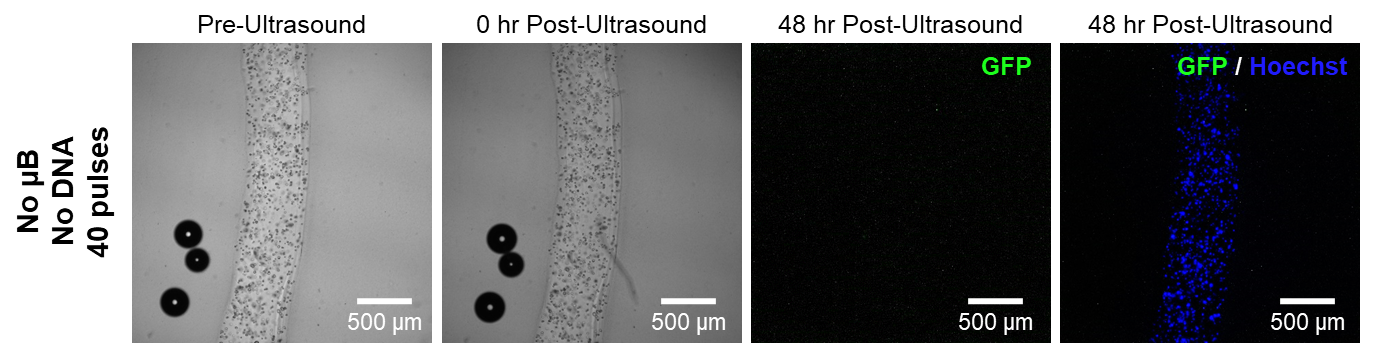


**Figure S6.** Representative images of coaxially-bioprinted osteoblast-laden (hFOB 1.19) 4% RGD-alginate bioink filament containing no microbubbles and no plasmid DNA, exposed to 40 ultrasound pulses. Filament shown in brightfield before and after ultrasound exposure, and via fluorescence microscopy at 48 hr post-ultrasound-exposure.
